# An *in vitro* evaluation of the effects of different statins on the structure and function of human gut bacterial community

**DOI:** 10.1371/journal.pone.0230200

**Published:** 2020-03-26

**Authors:** Changhui Zhao, Yunfei Hu, Huahai Chen, Baiyuan Li, Linyan Cao, Jinlan Xia, Yeshi Yin

**Affiliations:** 1 Key Lab of Biometallurgy of the Ministry of Education of China, School of Minerals Processing and Bioengineering, Central South University, Changsha, Hunan, China; 2 Key Laboratory of Comprehensive Utilization of Advantage Plants Resources in Hunan South, College of Chemistry and Bioengineering, Hunan University of Science and Engineering, Yongzhou, Hunan, China; State Key Laboratory for Diagnosis and Treatment of Infectious Diseases, CHINA

## Abstract

Statins, a class of drugs that can effectively remove cholesterol from serum, are used to regulate plasma total cholesterol and reduce the risk of cardiovascular diseases, but it is still unclear whether the drug are modulated by gut microbiota or the structures of gut microbiota are shaped by statins. We investigated the interactions between statins and the human gut microbiota during the *in vitro* fermentation process by *16S rRNA* gene sequencing, gas chromatography (GC), and high-performance liquid chromatography (HPLC) analyses. The presence of fluvastatin (FLU2) specifically promoted the growth of *Escherichia*/*Shigella*, *Ruminococcaceae* UCG 014, and *Sutterella*. However, the composition of the gut bacterial microbiota remained relatively static in samples treated with rosuvastatin (ROS), simvastatin (SIM), and atorvastatin (ATO). The PICRUSt program predicted moderate differences in the functional categories related to the biosynthesis of other secondary metabolites, cellular processes and signaling, and signal transduction in the FLU2 fermentation samples. Our study revealed substantial variation in the structure and function of microbiomes from the FLU2-treated samples. In addition, short-chain fatty acids (SCFAs) were also significantly decreased in FLU2-treated samples compared with the samples treated with other stains. Statins can be degraded by the human gut microbiota *in vitro*, and the degradation rate was approximately 7%–30% and 19%–48% after fermentation was allowed to proceed for 24 h and 48 h, respectively. Generally, FLU2 could largely shape the composition and function of human gut microbiota, which resulted in changes in the production of SCFAs. In turn, all statins could be degraded or modified by the gut microbiota. Our study paves the way for elucidating statin-gut microbiota interactions *in vitro* towards the improvement of the host health and personalized medicine.

## Introduction

The gastrointestinal tract, the largest digestive and excretive organ in the human body, is colonized by a vast, complex, and dynamic consortium of microorganisms [1]. It becomes clear that the human gut microbiota and host mutually affect and depend on each other in an intimate relationship [2]. Gut microbiota compositions can influence many aspects of the host [3, 4], including metabolism, obesity, maturation, regulation of the immune system, and even brain function and decision-making. The dysbiosis of gut microbiota may have negative impacts on health and eventually lead to diseases.

To gain insight into the relationship among gut microbiota drugs and human health, it is necessary to disentangle the structure, diversity, and function of the gut microbiota. A previous study using [5] *16S rRNA* gene profiling revealed that *Bacteroidetes* and the *Firmicutes* constitute over 90% of the known phylogenetic categories of the human gut bacterial microbiota [6]. Other studies showed that a substantial gut microbiome diversity exists between healthy individuals. However, there is a wide array of shared “core microbiome” and “core microbial species” among the sampled population [6, 7]. An increasing number of reports have shown that imbalances in the gut microbiota may cause intestinal dysfunctions and pathological states [8]. Furthermore, several studies on the impact of classes of drugs, xenobiotics, and dietary plant substances on the composition of the gut microbiota have been published [9]. Biocides, metals, and non-antibiotic chemicals with antibacterial properties also contributed to antimicrobial resistance via co-selection of resistant genes [10]. For example, Le Bastard et al. [11] thought that non-antibiotic prescription drugs have a notable impact on the gut microbiota composition. The disposition, efficacy, and toxicity of drugs that affect the gut microbiota could be explored by new sequencing and pyrotagging technologies [12]. Recent works showed that the gut microbiota could influence the pharmacokinetics of orally administered drugs and may have significant implications for their oral bioavailability [13].

Of the orally-administered lipid-lowering drugs developed to reduce the risk of cardiovascular disease, statins, which are 3-hydroxy-3-methylglutaryl-coenzyme A reductase inhibitors, are most commonly used worldwide. However, the long-term use of statins can cause a series of diseases, such as cytotoxicity, liver injury or necrosis, kidney damage, and myopathy [14]. It has been reported that gut microbiota play roles in the mediation of lovastatin metabolism and consequent pharmacokinetics interactions [15]. The hypolipidemic effects of SIM [16], ROS [17], and ATO [18] were shown to be correlated with the compositions of mice gut microbiota. Statins have certain antibacterial functions and have been described as novel adjuvant antibiotics [10], but we know little about the statin effects on the human gut bacterial microbiota. Hence, we investigated the interactions of SIM, FLU, ROS, and ATO with gut microbiota using *16S rRNA* gene high-throughput sequencing, GC, and HPLC analyses. Our aims were to discover if there are changes in the gut bacterial microbiota and KEGG pathways induced by different statins, statins are degraded or modified by gut microbiota, and whether the data would provide an explanation for the potential effects of statins on human health.

## Material & methods

### Ethical approval

All procedures performed in studies involving human participants were in accordance with the ethical standards of the Hunan University of Science and Engineering research committee and the 1964 Helsinki declaration (and its latest amendments) or comparable ethical standards.

### Statins

FLU, ROS, SIM, and ATO (pharmaceutical secondary standard, certified reference material) were purchased from Sigma-Aldrich Co. (St. Louis, MO, USA) and used as received, without further purification.

### Fecal sample collection

Fourteen healthy volunteers aged 18–22 years and living in Yongzhou city, Hunan province, China, were recruited for this study. Detailed information on these volunteers is shown in Table A in S1 Text. Participants did not receive antibiotics, probiotic, or prebiotic within the preceding three months. All donors provided informed consent, and the study was approved by the Ethics Committee of Hunan University of Science and Engineering (Yongzhou, China). Fecal samples were collected as soon as possible, and a fresh fecal sample (1 g) was immediately transferred to 10 mL of 0.1 M anaerobic PBS (pH 7.0) into glass beakers. Glass rods were then used to prepare a 10% (W/V) slurry, and then the solution was filtered through a four-layer gauze. The filtered slurries were used to inoculate the batch culture fermentation tubes; all of these steps were conducted in an anaerobic chamber except for fecal collection. The remainder was stored at -80°C for further analyses.

### *In vitro* batch culture fermentation

Batch culture fermentation was carried out using the procedure described by Hu et al. [19], Rycroft et al. [20], and Lei et al. [21]. The VI growth medium was adjusted to pH 6.5 with 1 M HCl, and starch (8 g/L) was prepared and added according to our previous studies [12, 19, 22]. The liquid culture media was autoclaved at 121°C for 15 min and cooled to room temperature under anaerobic conditions (10% H_2_, 10% CO_2_, and 80% N_2_). Statin was dissolved in 50% DMSO-50% water (v/v) and added to the medium to obtain a final statin concentration of 20 μM or colon concentration [23]; controls were added with 50% DMSO only (all samples content 1% DMSO finally). The colon concentrations of ROS, SIM, FLU, and ATO were set at the dose of 10 mg, 20 mg, 80 mg, and 20 mg per day [24], which is about 30, 40, 240, and 64 μg/mL *in vitro*, and we detected the concentrations at 0 h to establish a baseline (28, 33, 265, and 82 μg/mL, respectively). The filtered fresh fecal slurry (500 μL) was added to the previously prepared fermentation culture medium (9.5 mL), within an anaerobic chamber, and then incubated at 37°C. Two-milliliter samples collected at 24 h and 48 h for further analyses.

### Extraction of bacterial genomic DNA

The genomic DNA was extracted using the QIAamp DNA Stool Mini Kit according to the manufacturer’s instructions, and the lysis temperature was increased to 95°C to promote gram-positive bacterial lysis (QIAGEN, Hilden, Germany). The concentration of extracted DNA was quantified by a spectrophotometer (Colibri Microvolume Spectrometer, Titertek Berthold, Pforzheim, Germany).

### *16S rRNA* gene analysis of gut microbiota

The V3–V4 region of bacterial *16S rRNA* genes was amplified using the primers 338F (5’-ACT CCT ACG GGA GGC AGC AG-3’) and 806R (5’-GGA CTA CHV GGG TWT CTA AT-3’) [25]. The amplicons were sequenced on the Illumina MiSeq 300PE platform by Majorbio Bio-Pharm Technology Co., Ltd. (Shanghai, China). The resulting reads were analyzed using the Quantitative Insights Into Microbial Ecology (QIIME 2) pipeline. Then high-quality sequences were taxonomically classified in defining operational taxonomic units (OTUs) using Mothur at 97% sequence similarity [26]. The representative sequences were chosen and classified using the RDP classifier method [27, 28] and aligned against the SILVA database (https://www.arb-silva.de/documentation/release-123/). Good’s coverage, α-diversity, Simpson and Shannon index, and richness (number of OTUs) were analyzed using Visual Genomics–AS software (http://amplicon.vgenomics.cn:9000/). The data have been deposited in the sequence read archive (SRA) of NCBI as GenBank Accession Number PRJNA562063. Basic data on *16S rRNA* gene high-throughput sequencing are listed in Table B in S1 Text.

PICRUSt [29] software was used in this study to predict the metagenome functional profiling by using the bacterial *16S rRNA* gene sequences. The distribution of the Kyoto Encyclopedia of Genes and Genomes (KEGG) pathways of these samples predicted by PICRUSt software was further used for statistical analysis.

### Short-chain fatty acids analysis

SCFAs were determined by GC, as previously described [30, 31]. Briefly, 1 mL of the fermentation products were mixed with 0.2 mL 25% (w/v) of metaphosphoric acid, prior to mixing, the metaphosphoric acid solution was added croconic acid as an internal standard (final concentration 10 mmol/L). The samples were frozen at -20°C overnight, and then subsequently centrifuged (14,000 g for 20 min). The supernatant was used for SCFAs analysis using a GC-2010 Plus (Shimadzu Corporation, Japan) equipped with a flame ionization detector and an InertCap FFAP (0.25 mm ×30 m × 0.25 μm) column. The peaks were integrated using GC Solution software, and SCFA content was quantified using the single-point internal standard method. Peak identity and internal response factors were determined using a 20-mM calibration cocktail that included acetic, propionic, isobutyric, butyric, isovaleric, and valeric acids. The chromatograms of these SCFA mixtures are shown in S1 Fig.

### Statin concentration analysis

Statins were analyzed by the Shimadzu Black 20 AT system (Shimadzu, Kyoto, Japan) as previously described [32, 33] with some modifications. Briefly, statin fermentation samples were just centrifuged (14,000 g for 20 min), and the supernatant was used for statin analysis by HPLC. Chromatographic software Lab Solution was used for data collection and processing. Statins were detected by a UV-vis diode array detector at 238 nm. A Vertex C18 analytical column (4.6×250 mm, 5.0 μm, Vertex, USA) was used for the HPLC separation of statins. The injection volume was 10 μL, and the column oven temperature was kept at 30°C. The binary mobile phase, which is composed of acetonitrile and 0.1% formic acid (65:35), was pumped at a flow rate of 1.0 mL/min. The chromatograms and standard curves of stains are shown in S2 Fig.

### Statistical analysis

SPSS software (version 20.0; SPSS Inc., Chicago, IL, USA) and the Tamhane’s T2 (M) test were used to perform statistical analyses. The p values were then used to compute the false discovery rate (FDR) to account for multiple hypothesis testing using Microsoft Excel. *P* values and FDR less than 0.05 were considered statistically significant. Plot cladograms and significantly different bacterial taxa were analyzed using LEfSe Software (https://bitbucket.org/biobakery/biobakery/wiki/lefse) with default parameters and statistical methods.

## Results

### *16S rRNA* gene sequencing and diversity analysis

In summary, 245,914,669 and 2,011,409,834 valid reads were obtained from 14 original fecal samples and 126 fermented samples. Reads that could be classified as bacteria (97% sequence identity) were used for the subsequent analysis. The rarefaction curves, Shannon-Wiener curves, specaccum analysis, and rank-abundance distribution curve were used to evaluate the sequence quality (S3 Fig), which indicated that the number of sequences tended toward saturation as a function of sequence depth, suggesting that most of the bacterial sequences were captured in all samples.

In this study, a total of 1,212 OTUs from 140 samples were obtained based on 97% similarity. For the original fecal samples, 146–276 OTUs were identified, 69–511, and 98–372 OTUs were identified in the statins and DMSO fermented samples, respectively. The indexes of Ace, Chao, Shannon, and Simpson were calculated for fully illustrating the α-diversity of the bacterial communities. The α-diversity indexes of Ace, Chao, and Shannon were slightly different among the original fecal, DMSO, ROS, ATO, FLU and SIM samples (Table B in S1 Text and S3 Fig).

**Fig 1:**
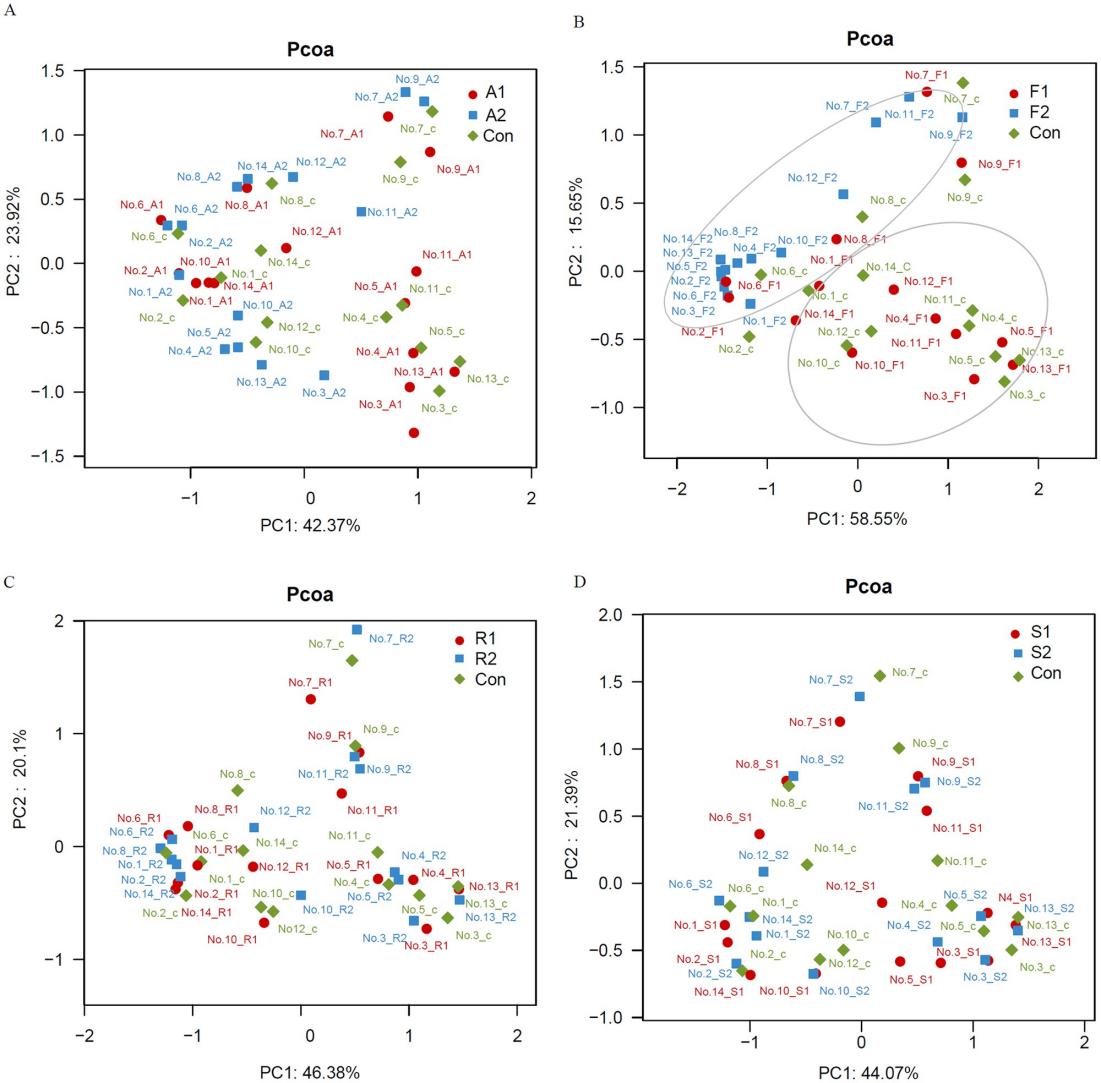
Principal coordinates analysis (PCoA) plot. PCoA plot of the gut microbiota based on the unweighted UniFrac metric. Basic analysis of sequenced data. Con, A1, A2, F1, F2, R1, R2, S1, and S2 represent the gut microbiota of fermented samples using the media plus DMSO and different statins at 20 μM and colon concentration. The p value of A, F, R, and S is 0.951, 0.006, 1, and 0.999, respectively, which was obtained by permanova.

As illustrated in Fig 1, β-diversity analysis was employed to elucidate the similarity of the samples used in this study. The results showed that the gut microbiota was most substantially affected by FLU2, followed by ATO2, SIM, and ROS. Most of the samples from groups FLU2 were clustered together, and separated from FLU1 and control samples.

### Effects of statin on the composition of human gut bacterial communities

**Fig 2:**
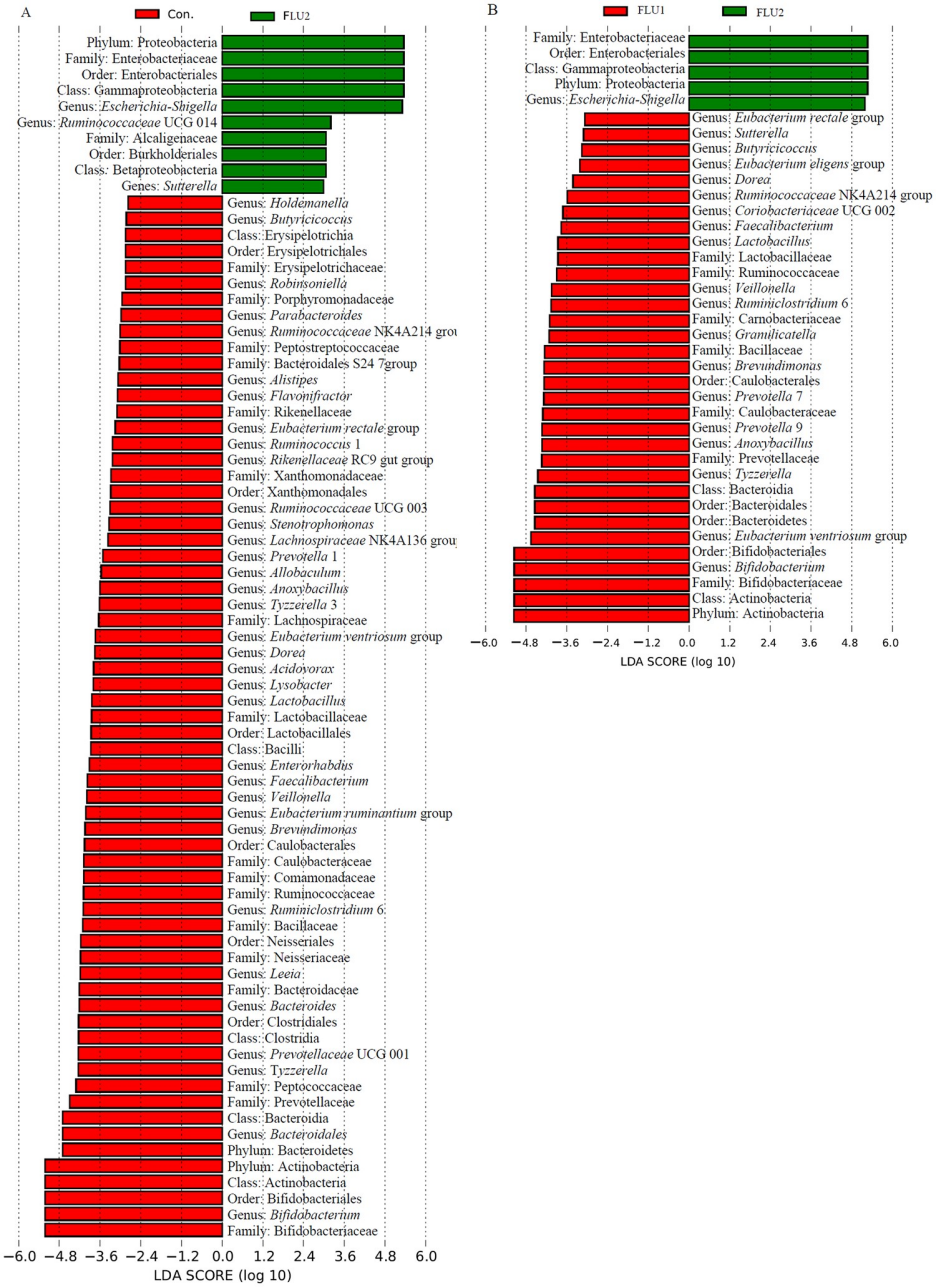
Analysis of the different abundant bacterial taxa using LEfSe. The bacterial percentage of the fermented samples was used for LEfSe analysis. The P-value <0.05 was identified as being significantly different between groups. Significantly enriched bacterial phyla, classes, orders, families, and genera are listed next to the histogram. Con, FLU1, and FLU2 represent samples collected after cultured using VI media with DMSO, 20 μM, and colon concentration of FLU. **A** Significantly different bacteria between FLU1 and FLU2. **B** Significantly different bacteria between groups of FLU2 and DMSO.

**Fig 3:**
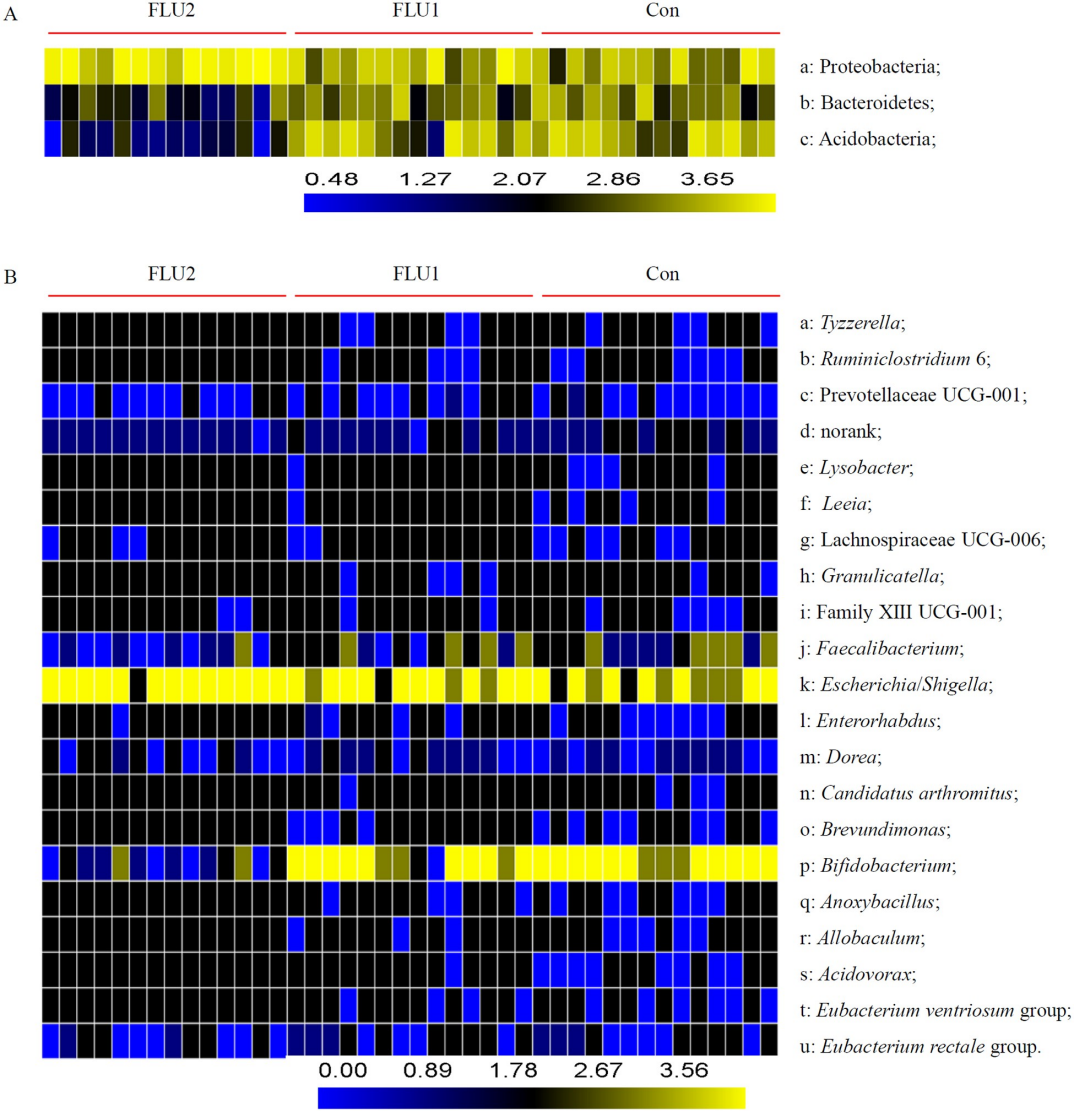
Relative abundance of individual gut microbiota composition at different taxonomic rank. Heatmap visualizes logarithm (base-10) of ratios (p<0.05). **A** Phyla found to be statistically different among the three groups. **B** Genera found to be statistically different among the three groups.

After classification, 19 bacterial phyla and 286 bacterial genera were identified from all samples (Table B in S1 Text). For the specific bacterial taxa among statin fermentation samples, we employed the linear discriminant analysis effect size (LEfSe) method and identified the significantly different bacteria across all taxa levels. The results showed that the addition of FLU2 and ATO2 statins significantly altered the structures of the gut bacterial microbiota. The phylum *Proteobacteria*, *Bacteroidetes*, and *Actinobacteria* were significantly affected by FLU2 when compared with groups FLU1 and DMSO (Figs 2 & 3A). The *Actinobacteria* were significantly affected by ATO2 compared with DMSO (S4 Fig).

At the genus level, the relative abundance of gut bacterial microbiota among all samples is shown in S5 Fig. *Escherichia/Shigella*, *Bifidobacterium*, *Megamonas*, *Prevotella* 9, *Megasphaera*, *Bacteroides*, *Klebsiella*, *Faecalibacterium*, *Lactobacillus*, *and Citrobacter* are the top 10 genera. *Escherichia/Shigella*, *Ruminococcaceae* UCG 014, and *Sutterella* were significantly enriched in groups FLU2 compared with DMSO, and *Bifidobacterium*, *Bacteroidales*, *Tyzzerella*, *Prevotellaceae* UCG 001, *Bacteroides*, *Leeia*, *Ruminiclostridium* 6, *Brevundimonas*, *Eubacterium ventriosum* group, *Veillonella*, *Enterorhabdus*, *Lysobacter*, *Acidovorax*, among others, were enriched in DMSO (Fig 2A). Only *Escherichia/Shigella* were enriched in FLU2 compared with FLU1, and *Bifidobacterium*, *Eubacterium ventriosum* group, *Tyzzerella*, *Anoxybacillus*, *Prevotella* 9, *Prevotella* 7, *Brevundimonas*, *Granulicatella*, *Ruminiclostridium* 6, among others, were enriched in FLU1 (Fig 2B). Some genera, such as *Bifidobacterium*, *Tyzzerella*, and *Lactobacillus*, were also decreased significantly in the ATO2 groups compared with DMSO groups (S4 Fig). However, those genera were not statistically affected by the colon concentration of other statins compared with DMSO or 20 μM statin.

### Predictive metagenome functional profiling of gut microbiota

**Fig 4:**
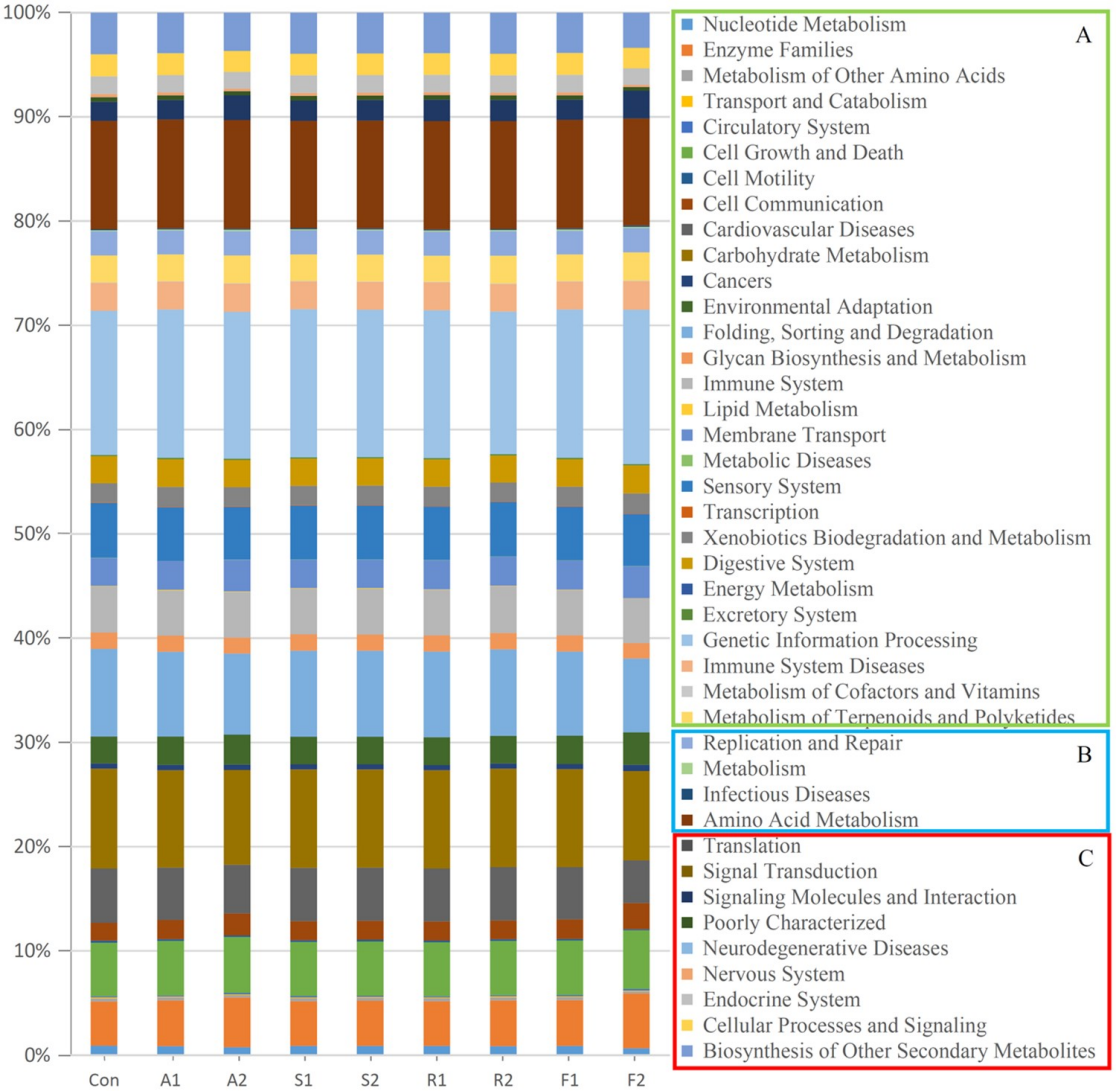
KEGG categories present in the human gut microbiota. The KEGG pathways in box A were not affected by FLU2; the KEGG pathways in boxes B and C were affected significantly by FLU2 compared with other statins (Box A p>0.05; Box B: p<0.05, 0.05<FDR<0.1; Box C: p<0.05, FDR<0.05).

**Fig 5:**
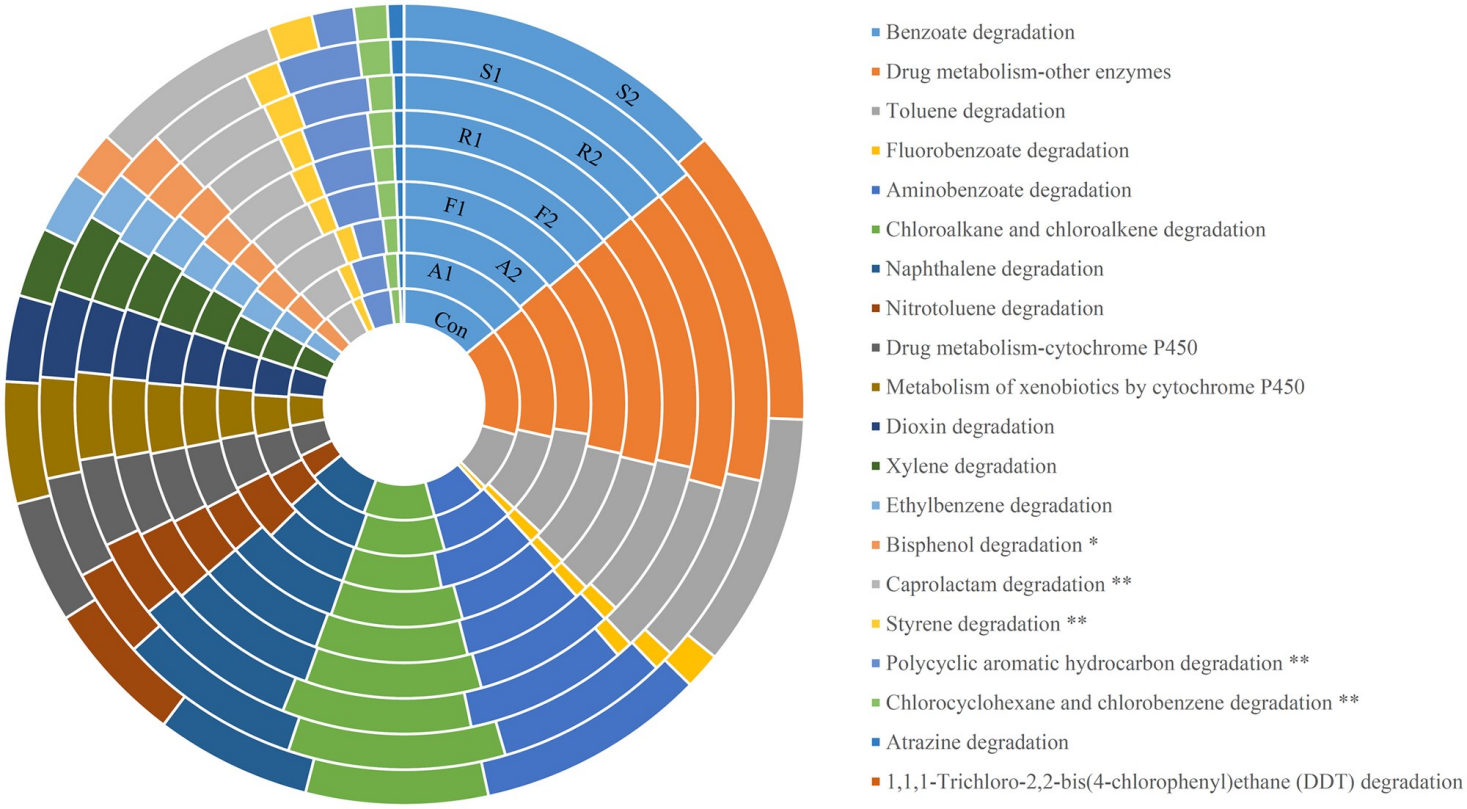
Functional analysis of the proteins involved in biosynthesis of xenobiotics biodegradation and metabolism. ** means significantly different between F2 and some other samples except for A2 (*p*<0.05; FDR<0.05); * means significantly different between F2 and some other samples except for A2 (*p*<0.05; 0.05<FDR<0.1).

For function annotation, PICRUSt [29] was used to predict the metagenome functional contents based on the *16S rRNA* gene sequences of the gut bacterial microbiota. These results showed that statins dramatically altered the abundance of many proteins encoded by the gut microbiota, particularly in the FLU2 samples. The functions of the genes that were changed more significantly (FDR<0.05) in FLU2 than in other statins, except for ATO2, as showed in the rectangular box C. For instance, the KEGG pathways of cellular processes and signaling, signal transduction, and neurodegenerative diseases, were significantly increased in FLU2; the pathways of biosynthesis of other secondary metabolites, endocrine system, nervous system, signaling molecules and interaction, were significantly decreased in FLU2. In box B, these pathways were altered more significantly (FDR<0.1) in FLU2 than others except for ATO2, the pathway of replication and repair was not significantly different between FLU2 and the control when considering FDR. In box A, it can be seen that some pathways were not affected by some statins or were affected but with an FDR>0.1 (Fig 4). Moreover, the xenobiotics biodegradation and metabolism pathway were further analyzed. The degradation of caprolactam, styrene, polycyclic aromatic hydrocarbon, chlorocyclohexane and chlorobenzene were found to be significantly affected by FLU2 when compared with other statins and the control (Fig 5).

### Concentration of SCFAs

**Fig 6:**
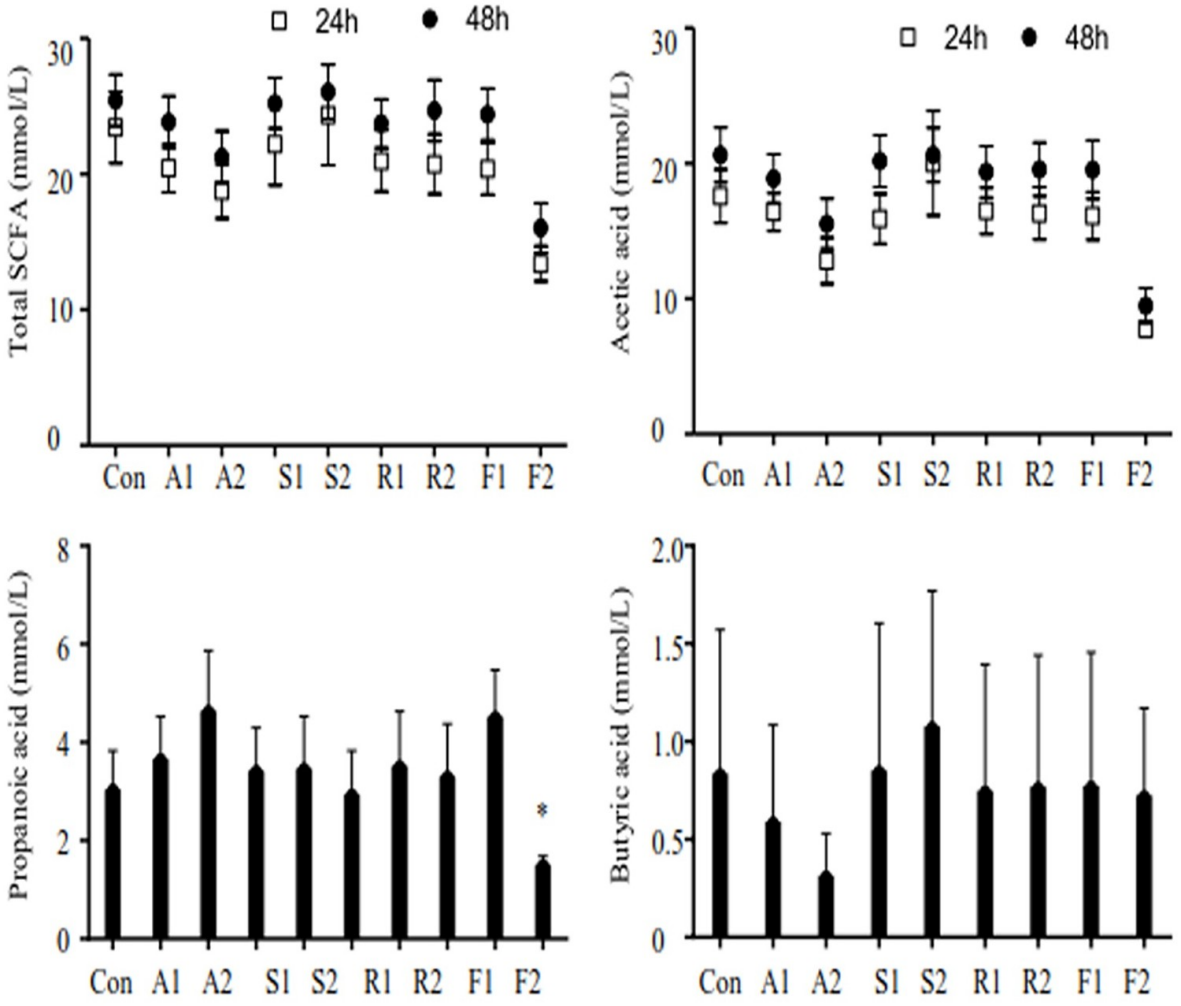
Effect of statins on SCFA production after fermentation. Acetic, propionic, isobutyric, butyric, isovaleric, and valeric acid were detected using GC in this paper. Each sample was measured in triplicate. Figures were generated using GraphPad Prism version 5.01. The figure shows total SCFA, acetic, propionic, and butyric acid concentration of the following samples: (1: 20 μM; 2: colon concentration; A: ATO; S: SIM; R: ROS; F: FLU).

After 24 h and 48 h fermentation, the concentration of total SCFAs, acetic, propionic, butyric acid in the fermentation samples were not significantly different, except for the acetic and propionic acid in FLU2 samples (p<0.05). The concentration of propionic and butyric acid was affected more in the ATO2 groups than in other groups (Fig 6).

### Degradation or modification of statin by human gut microbiota

**Fig 7:**
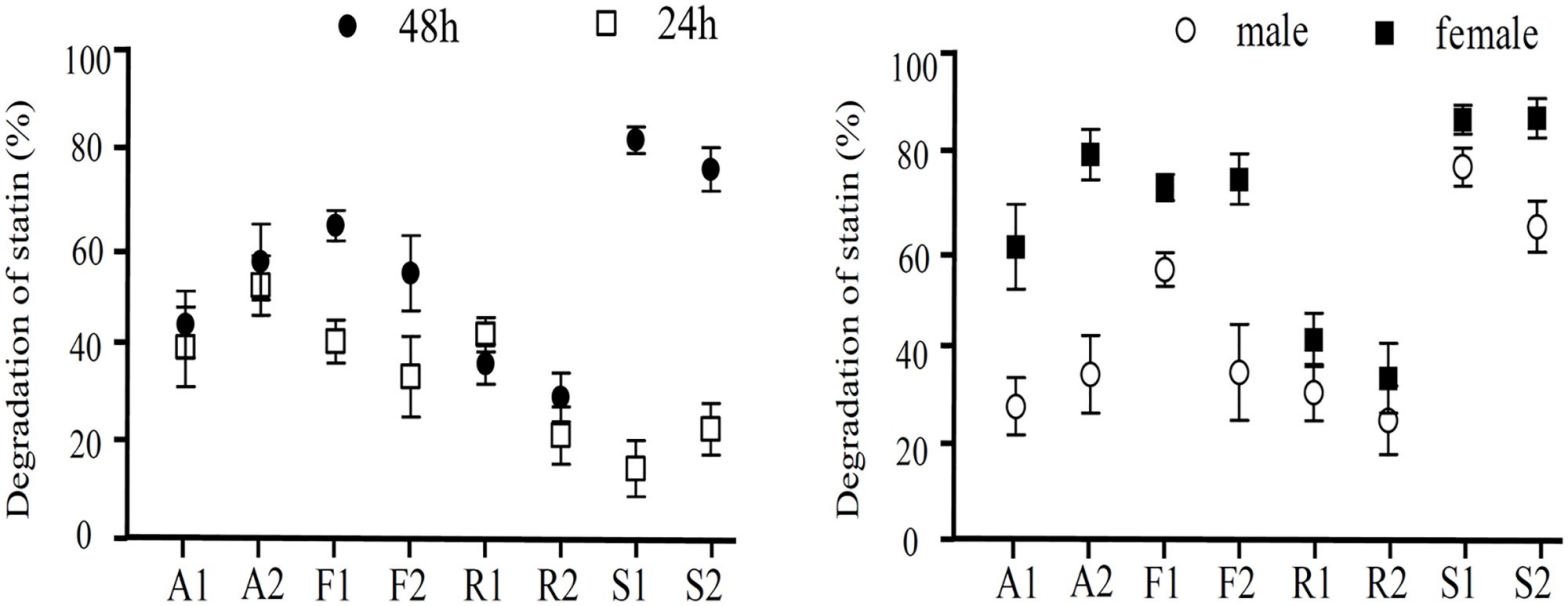
Degradation of statins. Different statins were detected by HPLC, and the degradation was obtained by the original percentage subtracted measurable percentage. Each statin sample was measured in duplicate.

To evaluate whether the detected concentration of stains was different from the expected calculation concentration, the concentrations of ROS, SIM, FLU, and ATO at 0 h were used as a baseline for the calculation of the degradation ratio. After fermenting for 24 h and 48 h, the degradation or modification rates of ATO by the human gut microbiota were a little higher in the colon concentration groups than in the 20 μM groups (Fig 7). The degradation or modification rates of SIM were up to 77.1%, and the minimum degradation rate of ROS was about 32.7%. However, 13.4%–24.1% of statins were precipitated in the culture medium or absorbed by the gut microbiota after high-speed centrifugation (data not shown); the real degradation or modification was approximately 7–30% and 19%-48% after fermentation for 24 h and 48 h, respectively. Compared with the male samples, the degradation rates of ATO and FLU were higher in the female samples.

## Discussion

The human body hosts a complex microbial ecosystem that facilitates host metabolism and adiposity by expanding nutrient sources, producing essential vitamins, and carrying out xenobiotic metabolism [34]. Moreover, xenobiotics, including therapeutic drugs, can potentially alter the gut microbiota community structures and associated functions [35]. However, relatively little is known about how the gut microbiota interacts with xenobiotics during long-term exposures [17]. Statins, the most effective lipid-lowering drugs, which are widely used for the treatment of hypercholesterolemia owing to their inhibition of HMGR protein [36], also have off-target effects including anti-inflammation function [37, 38]. The antibacterial activities of these drugs have been widely reported but little is known about their effects on gut microbiota. Our experiment showed that *Bacteroidetes* decreased significantly in FLU2 fermentation samples, and numerous studies [3, 39, 40] found that the obesity is associated with changes in the relative abundance of the *Bacteroidetes*, not only in mice but also in human. For instance, previous research with germ-free mice showed that obesity decreased the relative abundance of *Bacteroidetes* [41]. Other studies [42, 43] have suggested that a significant reduction of the phylum *Bacteroidetes* occurs in Crohn’s disease/ulcerative colitis patients compared with controls. The meta-analysis also showed a significant lowering of LDL, and increases in height and weight with statin administration [44]. All of these data might imply that a high dose of FLU may cause obesity, disease, or intestinal microbiota disorders, at least partially.

Considering that the VI medium could simulate the human gut microbiota *in vitro* with higher than 80% similarity with the original fecal bacterial community [22], VI medium was used in this study. After fermentation *in vitro*, the high throughput sequencing results showed that the FLU2 samples were enriched for the microbial groups of *Escherichia/Shigella*, *Ruminococcaceae* UCG 014, and *Sutterella*. Given the higher relative abundance of *Escherichia/Shigella*, the gut microbiota would be more susceptible to colorectal cancer [45]. However, some particular drugs exert their therapeutic effects through selectively altering the structure of the gut microbiota, for example, increasing the abundance of *Escherichia* [46]. *Sutterella* is an opportunistic bacterium of the phylum *Proteobacteria*; human who have higher levels of the genera of *Sutterella* would have a lower concentration and percentage of butyric acid [47]. In particular, there is evidence that the *Sutterella* species is associated with autism, Down’s syndrome, and inflammatory disease [48, 49]. Menni and collaborators [50] found that arterial stiffness is negatively correlated with the abundance of the family *Ruminococcaceae*. It is assumed that the effects of statins are largely individual-dependent. Specifically, the family *Ruminococcaceae* was enriched differently by statins. However, Scheperjans et al. [51] found that the *Ruminococcaceae* population was increased significantly in Parkinson’s disease (PD) compared with healthy controls. Based on those analyses, our research could explain why the use of statin is associated with a higher risk of PD [52]. Notably, the family *Ruminococcaceae* was also significantly increased in patients with constipation [53].

After treating the gut microbiota with statins, there was a significant reduction of several clusters, including the genus *Bifidobacterium* in the FLU2 fermentation samples compared with the other samples. These differences between FLU2 and other statins could be explained by the high doses and low lipid-lowering effect of FLU. Taken together, patients that need to reduce LDL-C levels more than 35% might choose ROS first, then ATO and SIM, and should not be given FLU. The patient’s statin-sensitive responses are linked to the gut microbiota, and the gut microbiota may guide the statin dosage adjustments [54]. There are indeed several reports showing that statins influence the diversity of the human gut microbiota [18, 55–57]. In addition, although the research on the antibacterial activity of FLU has been scarce and more studies are needed [58], it seems that its antifungal activity may be higher than that of other statins [59]. In another study, administration of ROS to mice randomly in the diet for 28 days significantly influenced the structure of the gut microbiota [17]. Some researchers found that ATO and SIM may have a more potent antibacterial activity than ROS, but the clinical strains were less sensitive to statin compared with the standard bacteria [60]. Thus, during the *in vitro* fermentation, the gut microbiota was not significantly affected by ROS, ATO, and SIM at a suitable dose. Hence, the differences between FLU2 and other samples might further support the findings that a high dose of statins is not adequate for Chinese people [61].

We did not observe significant differences in bacterial diversity between two concentrations of the same statins (except for FLU), but differences were detected for different statins. This suggested that the gut microbiota was influenced differently by different statins and that a variation of the microbiota occurs much like the ecological successions associated with the genetics, social environment, diet, metabolic disorders, and antibiotic treatment [62]. However, some research works provide evidence that there is little “stability” within the resident clonal populations of the common gut bacterial family of *Enterobacteriaceae*. Given that clones can vary substantially in genome content and that evolutionary processes operate at the population level, the biological relevance of our results may require the demonstration of stability at lower taxonomic levels [63].

PICRUSt allowed us to know more about the overall microbiome metabolism and its functional specialization. We also performed a statistical analysis of differences in all samples. Interestingly, about 50.91% KEGG pathways were significantly changed in FLU2 compared with controls, and 10.67% KEGG pathways were significantly affected in ATO2, but there were no statistical differences in ROS and SIM when compared with controls. Consistently, the content of predictive metagenome functionalities was remarkably influenced by FLU2 when compared with other statins. FLU2 only slightly affected xenobiotics biodegradation and metabolism, but the effect was statistically different in some specific substances. For example, caprolactam degradation and styrene degradation were significantly overrepresented in the FLU2 metagenome. Those pathways may be related to FLU degradation, and further studies are required for elucidating the pharmacological or toxicological properties of the gut microbial metabolites and metabolites contribution during statin treatment.

From another perspective, very few studies have examined how the human gut microbiota degrades statins. We are aware of only two studies that have considered the degradation of statin by gut microbiota. One study, based on the fecal samples from nine healthy volunteers, found that β-hydroxy acid form lovastatin was degraded by the gut microbiota *in vitro* [64]. Likewise, a study reported the degradation of SIM by the human gut microbiota, and implicated the pathway of colonic microbial metabolism in this process [65]. However, the characteristics of the gut microbiota-driven degradation of statins have not yet been fully investigated, and a more thorough investigation is needed to understand the association between statins, the gut microbiota, and the host. Additional time points, metabolites of statins, and culture assays to determine if bacteria change in relative abundance need to be reported. The currently underdeveloped studies on the concentration change of statins after incubation with the human gut microbiota could become an exciting area of pharmacological research.

## Conclusion

We observed that FLU2 significantly influenced the composition of the gut bacterial microbiota and the functional profiles, and that it changed SCFA production correspondingly. In turn, the statins were also degraded or modified by gut microbiota. The results may provide support for the microbial mediation of the therapeutic effects of FLU through short-chain fatty acid production, as well as for potential microbiota-mediated mechanisms behind the known intestinal adverse effects in the form of a relative increase in abundance of *Escherichia/Shigella*, *Ruminococcaceae*, and *Sutterella*. It is unclear whether the statistically significant alterations in gut microbiota have the potential for promoting key physiological adaptations and lipid-lowering effects. Further studies are needed to evaluate whether the gut microbiota alters the disposition, toxicity, efficacy of statins, and the analyses on interactions among drugs, gut microbiota, SCFA, and host should be extended to more populations and cover longer periods. In this myriad of untapped interactions, one may find strategies for personalized medicine to improve health and longevity.

## Supporting information

S1 Text Table A. Pharmacokinetic parameters of used statins. Table B. Basic statistical results for high-throughput 16S rRNA gene sequencing.
(DOCX)

S1 Fig Chromatograms of standard SCFAs and crotonic acid. (PDF)

S2 Fig Chromatograms and standard curves of statins. (PDF)

S3 Fig Basic analysis of sequenced data. (PDF)

S4 Fig LEFSe analysis of different abundant bacterial taxa between groups ATO2 and control. (PDF)

S5 Fig Bacterial community abundance at the genus level. (PDF)
