# Supplementary material for: An in vitro evaluation of the effects of different statins on the structure and function of human gut bacterial community
Source: PLoS One. 2020 Mar 26;15(3):e0230200. doi: 10.1371/journal.pone.0230200 (PMC7098552; doi:10.1371/journal.pone.0230200)
Supplement: S1 Text — Table A. Pharmacokinetic parameters of used statins. Table B. Basic statistical results for high-throughput 16S rRNA gene sequencing. (DOCX) [file pone.0230200.s001.docx]

**Table A Pharmacokinetic parameters of used statins.**

| Statin | Molecular weight | Origin | Absorption (%) | Bioavailability (%) | | T_1/2_  (h) | Hepatic/Kidney excretion (%) | | Range of dose (mg/d) | 50% inhibit concentration (μg/mL) |
| --- | --- | --- | --- | --- | --- | --- | --- | --- | --- | --- |
| Simvastain (SIM) | 418.15 | Semi-synthetic | 65-85 | <5 | | 3 | 60/13 | | 5-80 | 7.6 |
| Rluvastatin (FLU) | 433.46 | Synthetic | 98 | 10～35 | | 3 | 90/6 | | 20-80 | 7.8 |
| Rosuvastatin (ROS) | 1001 | Synthetic | 50 | 20 | | 19 | 90/10 | | 5-80 | 12 |
| Atorvastatin (ATO) | 1209 | Synthetic | >70 | 12 | 11～30 | | | 98/2 | 10-80 | 18.4 |

T_1/2_: half-life of its metabolites in liver.

**Table B Basic statistical results for high-throughput 16S rRNA gene sequencing.**

| Samples ID | Age(years) | Sex | Reads | OUT(0.97) | ace | chao | shannon | simpson | coverage |
| --- | --- | --- | --- | --- | --- | --- | --- | --- | --- |
| No.1 | 19 | Male | 44581 | 224 | 377.5807 | 322.3077 | 2.969094 | 0.116113 | 0.997449 |
| No.1_A1 |  |  | 32855 | 165 | 342.0838 | 284.8947 | 1.713174 | 0.344391 | 0.99759 |
| No.1_A2 |  |  | 45714 | 118 | 210.103 | 178.2 | 1.031753 | 0.621598 | 0.998476 |
| No.1_F1 |  |  | 34383 | 131 | 239.5211 | 181.6471 | 1.685302 | 0.330947 | 0.998512 |
| No.1_F2 |  |  | 31106 | 133 | 296.6679 | 248.9091 | 1.484306 | 0.497209 | 0.998193 |
| No.1_R1 |  |  | 31926 | 164 | 221.9445 | 219.12 | 1.748019 | 0.330445 | 0.998122 |
| No.1_R2 |  |  | 30371 | 153 | 285.6154 | 204.4839 | 1.16318 | 0.466594 | 0.99798 |
| No.1_S1 |  |  | 31505 | 126 | 182.1652 | 167.1304 | 1.02982 | 0.49956 | 0.998441 |
| No.1_S2 |  |  | 45012 | 129 | 253.4648 | 223 | 1.564569 | 0.360341 | 0.998299 |
| No.1_c |  |  | 32405 | 134 | 245.5497 | 206.0667 | 1.713235 | 0.332377 | 0.998334 |
| No.2 | 20 | Male | 31917 | 167 | 213.376 | 227.8824 | 1.216866 | 0.622511 | 0.99837 |
| No.2_A1 |  |  | 30296 | 94 | 240.2292 | 164.7143 | 0.536673 | 0.764482 | 0.998405 |
| No.2_A2 |  |  | 32948 | 107 | 239.5717 | 209.2143 | 0.314151 | 0.89733 | 0.998086 |
| No.2_F1 |  |  | 31972 | 103 | 367.5847 | 236 | 0.457788 | 0.826664 | 0.99798 |
| No.2_F2 |  |  | 31265 | 69 | 188.7017 | 126.2727 | 0.097934 | 0.977808 | 0.998724 |
| No.2_R1 |  |  | 31508 | 96 | 144.1795 | 131.2857 | 0.601658 | 0.718218 | 0.998618 |
| No.2_R2 |  |  | 37987 | 102 | 312.1777 | 183.0588 | 0.469642 | 0.806617 | 0.998122 |
| No.2_S1 |  |  | 31162 | 88 | 185.4374 | 149.5 | 0.494113 | 0.773694 | 0.998512 |
| No.2_S2 |  |  | 33738 | 94 | 153.6071 | 163.4615 | 0.639846 | 0.690598 | 0.998476 |
| No.2_c |  |  | 31956 | 98 | 222.3261 | 145.8333 | 0.738444 | 0.648112 | 0.998512 |
| No.3 | 20 | Male | 32746 | 243 | 334.9182 | 294.1071 | 3.602133 | 0.049588 | 0.998086 |
| No.3_A1 |  |  | 32208 | 309 | 399.1304 | 394.0667 | 3.026149 | 0.104223 | 0.996882 |
| No.3_A2 |  |  | 35560 | 266 | 430.6647 | 341.6222 | 2.899442 | 0.117695 | 0.997059 |
| No.3_F1 |  |  | 42579 | 219 | 359.2831 | 292.7 | 2.747407 | 0.133965 | 0.997626 |
| No.3_F2 |  |  | 35335 | 219 | 418.6557 | 335.9063 | 0.887586 | 0.755023 | 0.996917 |
| No.3_R1 |  |  | 50372 | 317 | 511.0168 | 467.4054 | 2.713948 | 0.157084 | 0.996244 |
| No.3_R2 |  |  | 40044 | 368 | 469.72 | 473.0196 | 3.087441 | 0.110919 | 0.996315 |
| No.3_S1 |  |  | 30236 | 332 | 448.332 | 416.4426 | 2.838721 | 0.13499 | 0.996385 |
| No.3_S2 |  |  | 33928 | 359 | 502.9369 | 501.3529 | 2.937643 | 0.130761 | 0.995712 |
| No.3_c |  |  | 31915 | 236 | 436.461 | 331.2941 | 2.562677 | 0.176361 | 0.99713 |
| No.4 | 18 | Female | 31853 | 169 | 212.2641 | 210.0455 | 1.476987 | 0.534735 | 0.998476 |
| No.4_A1 |  |  | 39168 | 212 | 394.9144 | 349.3913 | 2.421431 | 0.183435 | 0.997165 |
| No.4_A2 |  |  | 39396 | 188 | 335.31 | 320.1429 | 1.860305 | 0.272675 | 0.997342 |
| No.4_F1 |  |  | 33230 | 295 | 414.9638 | 413.6957 | 2.191195 | 0.198322 | 0.996279 |
| No.4_F2 |  |  | 31522 | 121 | 332.226 | 235.8824 | 0.809807 | 0.571786 | 0.997767 |
| No.4_R1 |  |  | 32113 | 292 | 392.1506 | 370.6207 | 2.080661 | 0.239792 | 0.996598 |
| No.4_R2 |  |  | 31946 | 240 | 329.2224 | 308.25 | 2.008419 | 0.251432 | 0.997236 |
| No.4_S1 |  |  | 30443 | 267 | 340.2545 | 329.5625 | 2.319468 | 0.215602 | 0.997236 |
| No.4_S2 |  |  | 42627 | 280 | 481.6996 | 393.75 | 2.227068 | 0.194986 | 0.996279 |
| No.4_c |  |  | 40071 | 301 | 423.6891 | 400.0577 | 2.464667 | 0.175225 | 0.996385 |
| No.5 | 19 | Female | 34036 | 187 | 290.9522 | 277.5882 | 1.171481 | 0.675807 | 0.998016 |
| No.5_A1 |  |  | 32761 | 511 | 642.9831 | 646 | 3.425256 | 0.098567 | 0.995181 |
| No.5_A2 |  |  | 35719 | 268 | 362.2181 | 333.6316 | 2.15407 | 0.234093 | 0.996917 |
| No.5_F1 |  |  | 33562 | 397 | 539.0979 | 545.5167 | 2.255558 | 0.315421 | 0.995251 |
| No.5_F2 |  |  | 32706 | 94 | 342.6559 | 241.3333 | 0.67141 | 0.716141 | 0.998157 |
| No.5_R1 |  |  | 34163 | 439 | 591.4495 | 589.2581 | 3.297968 | 0.095162 | 0.995145 |
| No.5_R2 |  |  | 45409 | 199 | 244.6297 | 233 | 2.155561 | 0.218918 | 0.998157 |
| No.5_S1 |  |  | 32075 | 409 | 566.9991 | 578.2167 | 2.501598 | 0.19998 | 0.994932 |
| No.5_S2 |  |  | 38760 | 400 | 498.2758 | 484.6418 | 2.542277 | 0.255999 | 0.996208 |
| No.5_c |  |  | 39923 | 372 | 491.5052 | 510.06 | 2.33543 | 0.270085 | 0.995818 |
| No.6 | 20 | Male | 38750 | 146 | 302.4668 | 273.5 | 2.410881 | 0.179715 | 0.998193 |
| No.6_A1 |  |  | 48026 | 150 | 389.0893 | 296 | 0.893181 | 0.573465 | 0.997413 |
| No.6_A2 |  |  | 43054 | 204 | 370.6119 | 294.6176 | 1.264189 | 0.476099 | 0.9972 |
| No.6_F1 |  |  | 50997 | 170 | 305.5691 | 235.2069 | 1.22746 | 0.557281 | 0.997803 |
| No.6_F2 |  |  | 44309 | 140 | 260.4854 | 205.0455 | 0.873452 | 0.683362 | 0.998086 |
| No.6_R1 |  |  | 42372 | 137 | 355.802 | 224.6522 | 0.967253 | 0.475752 | 0.997732 |
| No.6_R2 |  |  | 35412 | 231 | 303.1067 | 321.0333 | 1.377196 | 0.444708 | 0.997378 |
| No.6_S1 |  |  | 40598 | 95 | 212.8458 | 181 | 0.845303 | 0.497882 | 0.998441 |
| No.6_S2 |  |  | 32173 | 253 | 380.9978 | 356.125 | 1.262593 | 0.461815 | 0.996456 |
| No.6_c |  |  | 37271 | 325 | 586.7807 | 523.7179 | 1.762959 | 0.389893 | 0.99557 |
| No.7 | 22 | Female | 32375 | 164 | 250.2428 | 233.1765 | 2.009947 | 0.243236 | 0.998264 |
| No.7_A1 |  |  | 31746 | 199 | 403.2599 | 319.2069 | 1.906854 | 0.28708 | 0.997023 |
| No.7_A2 |  |  | 34112 | 101 | 221.6711 | 181.5714 | 1.437281 | 0.347879 | 0.998299 |
| No.7_F1 |  |  | 36829 | 81 | 153.9352 | 123.2727 | 1.315435 | 0.44807 | 0.998901 |
| No.7_F2 |  |  | 32097 | 194 | 677.7354 | 384.7813 | 1.579982 | 0.280061 | 0.996066 |
| No.7_R1 |  |  | 42634 | 196 | 520.8753 | 370.84 | 1.719616 | 0.354662 | 0.996669 |
| No.7_R2 |  |  | 38072 | 96 | 137.7926 | 120.6667 | 0.894434 | 0.663306 | 0.998689 |
| No.7_S1 |  |  | 36781 | 121 | 321.2501 | 200.8 | 1.684359 | 0.302825 | 0.99798 |
| No.7_S2 |  |  | 42160 | 156 | 294.2201 | 236.4545 | 1.742191 | 0.34635 | 0.997874 |
| No.7_c |  |  | 46701 | 235 | 346.8917 | 339.65 | 1.599612 | 0.449972 | 0.99674 |
| No.8 | 20 | Male | 51265 | 201 | 295.2148 | 263.6667 | 3.043905 | 0.141036 | 0.998299 |
| No.8_A1 |  |  | 39789 | 159 | 227.2905 | 233.3913 | 1.544466 | 0.304783 | 0.997909 |
| No.8_A2 |  |  | 48572 | 236 | 446.7764 | 384.5294 | 1.677929 | 0.26428 | 0.996421 |
| No.8_F1 |  |  | 45226 | 212 | 338.5786 | 339.5714 | 1.628617 | 0.311089 | 0.996633 |
| No.8_F2 |  |  | 59503 | 174 | 346.2382 | 283.6154 | 1.257112 | 0.447304 | 0.997307 |
| No.8_R1 |  |  | 46652 | 144 | 233.3173 | 189.2308 | 1.702449 | 0.276271 | 0.998264 |
| No.8_R2 |  |  | 46834 | 156 | 254.5906 | 224.1429 | 1.672778 | 0.319987 | 0.998086 |
| No.8_S1 |  |  | 45891 | 178 | 336.3812 | 245.1622 | 1.400259 | 0.369738 | 0.997484 |
| No.8_S2 |  |  | 52359 | 178 | 418.4472 | 292.5172 | 1.589031 | 0.305036 | 0.997094 |
| No.8_c |  |  | 39380 | 181 | 374.0397 | 292.7241 | 1.599785 | 0.304299 | 0.99713 |
| No.9 | 19 | Male | 44245 | 189 | 351.2822 | 270.375 | 2.341218 | 0.240555 | 0.997767 |
| No.9_A1 |  |  | 30333 | 187 | 557.0189 | 370.3333 | 1.666399 | 0.348139 | 0.996456 |
| No.9_A2 |  |  | 42421 | 94 | 193.6064 | 151.4 | 1.358574 | 0.378462 | 0.998512 |
| No.9_F1 |  |  | 35862 | 101 | 232.8489 | 170.4615 | 1.846075 | 0.227115 | 0.998476 |
| No.9_F2 |  |  | 41902 | 142 | 489.0642 | 263.5385 | 1.097974 | 0.47644 | 0.997165 |
| No.9_R1 |  |  | 38341 | 104 | 243.4108 | 177.9286 | 1.908035 | 0.218608 | 0.99837 |
| No.9_R2 |  |  | 40047 | 98 | 221.1577 | 141.1579 | 2.104705 | 0.163533 | 0.998547 |
| No.9_S1 |  |  | 37880 | 279 | 514.6842 | 440.3333 | 2.11404 | 0.206973 | 0.995712 |
| No.9_S2 |  |  | 47909 | 166 | 324.5895 | 241.6774 | 1.96859 | 0.219057 | 0.997555 |
| No.9_c |  |  | 56848 | 224 | 482.436 | 360.4865 | 1.961923 | 0.240927 | 0.996421 |
| No.10 | 20 | Male | 43171 | 186 | 321.1457 | 248.2174 | 3.009461 | 0.10192 | 0.998086 |
| No.10_A1 |  |  | 33400 | 143 | 360.0554 | 270.1053 | 1.626281 | 0.349751 | 0.997519 |
| No.10_A2 |  |  | 30608 | 205 | 289.3762 | 267.3415 | 1.927936 | 0.268543 | 0.997449 |
| No.10_F1 |  |  | 41626 | 254 | 637.9255 | 504.3448 | 2.208103 | 0.20889 | 0.995712 |
| No.10_F2 |  |  | 38050 | 217 | 408.5152 | 312.9231 | 1.413525 | 0.410878 | 0.996917 |
| No.10_R1 |  |  | 32442 | 157 | 279.1538 | 216.0357 | 1.93618 | 0.245506 | 0.997945 |
| No.10_R2 |  |  | 39462 | 141 | 256.6137 | 194 | 2.139423 | 0.171847 | 0.998122 |
| No.10_S1 |  |  | 41164 | 224 | 499.0613 | 504.5556 | 2.002019 | 0.252877 | 0.996421 |
| No.10_S2 |  |  | 34406 | 151 | 188.924 | 178.3333 | 2.017397 | 0.242181 | 0.998547 |
| No.10_c |  |  | 35563 | 233 | 420.4779 | 343.1579 | 2.193657 | 0.209805 | 0.99674 |
| No.11 | 19 | Female | 38627 | 200 | 304.2054 | 297.2353 | 1.484034 | 0.545293 | 0.997945 |
| No.11_A1 |  |  | 34900 | 233 | 469.7856 | 392.6774 | 2.372906 | 0.145087 | 0.996456 |
| No.11_A2 |  |  | 35527 | 217 | 372.3819 | 318.9355 | 2.260806 | 0.180408 | 0.997165 |
| No.11_F1 |  |  | 34424 | 218 | 377.7131 | 317.1071 | 2.4294 | 0.156314 | 0.997342 |
| No.11_F2 |  |  | 35486 | 102 | 213.9102 | 184.5 | 1.500566 | 0.327096 | 0.998405 |
| No.11_R1 |  |  | 44706 | 201 | 333.1744 | 292.12 | 2.442454 | 0.156993 | 0.99759 |
| No.11_R2 |  |  | 46583 | 221 | 453.4409 | 316.0667 | 1.643835 | 0.429977 | 0.996704 |
| No.11_S1 |  |  | 40552 | 213 | 384.9944 | 309.2813 | 2.391587 | 0.150265 | 0.9972 |
| No.11_S2 |  |  | 40894 | 210 | 379.8764 | 312.7 | 2.429011 | 0.149708 | 0.9972 |
| No.11_c |  |  | 34070 | 219 | 300.6472 | 296.6563 | 2.524233 | 0.135709 | 0.997484 |
| No.12 | 17 | Female | 57463 | 276 | 413.8989 | 349.15 | 3.122574 | 0.093833 | 0.997271 |
| No.12_A1 |  |  | 33732 | 198 | 406.6005 | 301.9167 | 1.804162 | 0.247433 | 0.996917 |
| No.12_A2 |  |  | 46568 | 172 | 498.0327 | 339.44 | 1.422861 | 0.360399 | 0.99674 |
| No.12_F1 |  |  | 50425 | 204 | 516.9797 | 348.0303 | 2.045962 | 0.201487 | 0.996527 |
| No.12_F2 |  |  | 31353 | 130 | 346.2157 | 224.5455 | 1.310375 | 0.357201 | 0.997697 |
| No.12_R1 |  |  | 31845 | 252 | 641.4328 | 436.525 | 1.90998 | 0.23527 | 0.995677 |
| No.12_R2 |  |  | 54048 | 248 | 544.3684 | 397.7907 | 1.858083 | 0.263291 | 0.99596 |
| No.12_S1 |  |  | 38189 | 344 | 651.0185 | 519.0556 | 2.642128 | 0.136178 | 0.99511 |
| No.12_S2 |  |  | 44267 | 119 | 228.2101 | 183.4737 | 1.443923 | 0.330826 | 0.998228 |
| No.12_c |  |  | 36560 | 252 | 389.8815 | 363.3 | 2.073128 | 0.211517 | 0.996244 |
| No.13 | 19 | Female | 32604 | 225 | 265.1316 | 259.871 | 3.217669 | 0.080757 | 0.998334 |
| No.13_A1 |  |  | 38848 | 182 | 345.351 | 287.2857 | 2.257151 | 0.215362 | 0.997626 |
| No.13_A2 |  |  | 38036 | 181 | 408.9883 | 270.0625 | 2.104846 | 0.2102 | 0.997307 |
| No.13_F1 |  |  | 37243 | 251 | 415.8189 | 381.5357 | 2.345751 | 0.229131 | 0.996952 |
| No.13_F2 |  |  | 33308 | 136 | 269.4755 | 220 | 0.572042 | 0.815783 | 0.99798 |
| No.13_R1 |  |  | 40607 | 256 | 458.8448 | 373.5 | 1.987482 | 0.319588 | 0.996633 |
| No.13_R2 |  |  | 38471 | 211 | 386.2803 | 308.5333 | 2.086597 | 0.281873 | 0.997271 |
| No.13_S1 |  |  | 37784 | 257 | 513.5794 | 383.25 | 2.052882 | 0.328377 | 0.996421 |
| No.13_S2 |  |  | 30891 | 235 | 457.8972 | 359.0278 | 2.186595 | 0.27604 | 0.996633 |
| No.13_c |  |  | 33412 | 201 | 380.9055 | 347.7143 | 2.111658 | 0.249502 | 0.9972 |
| No.14 | 18 | Female | 50771 | 163 | 210.3465 | 225.6667 | 1.532133 | 0.497935 | 0.998299 |
| No.14_A1 |  |  | 31587 | 193 | 508.9375 | 354.8889 | 1.619887 | 0.318159 | 0.996669 |
| No.14_A2 |  |  | 33929 | 129 | 283.8339 | 211.65 | 1.535445 | 0.28576 | 0.997945 |
| No.14_F1 |  |  | 33841 | 294 | 443.9275 | 453.6383 | 1.880652 | 0.306696 | 0.995641 |
| No.14_F2 |  |  | 36502 | 129 | 219.9217 | 218.375 | 0.752678 | 0.662343 | 0.997661 |
| No.14_R1 |  |  | 35502 | 178 | 369.7398 | 265.5676 | 1.523397 | 0.372098 | 0.99713 |
| No.14_R2 |  |  | 34603 | 162 | 247.6491 | 258.6 | 1.47344 | 0.32815 | 0.997519 |
| No.14_S1 |  |  | 37582 | 272 | 507.1217 | 409.0426 | 1.256242 | 0.514055 | 0.99596 |
| No.14_S2 |  |  | 34779 | 310 | 464.082 | 446.6852 | 1.789971 | 0.311427 | 0.995677 |
| No.14_c |  |  | 32292 | 193 | 479.8164 | 416.25 | 1.803271 | 0.238363 | 0.996633 |
