# Supplementary material for: An in vitro evaluation of the effects of different statins on the structure and function of human gut bacterial community
Source: PLoS One. 2020 Mar 26;15(3):e0230200. doi: 10.1371/journal.pone.0230200 (PMC7098552; doi:10.1371/journal.pone.0230200)
Supplement: S1 Fig — (PDF) [file pone.0230200.s002.pdf]

S1 Fig Chromatograms of standard SCFAs and crotonic acid.

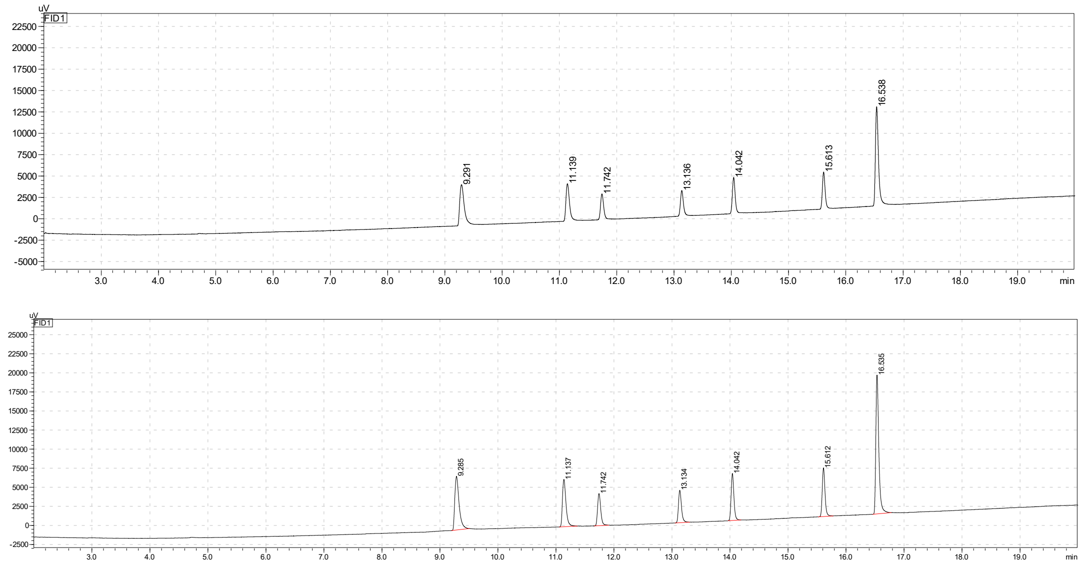

Notes:

Acetic acid: 9.291 min, LOD=0.2187, LOQ= 0.7290 mmol/L;

Propanoic acid: 11.137 min, LOD=0.0806, LOQ= 0.2687 mmol/L;

Isobutyric acid: 11.742 min, LOD=0.1589, LOQ=0.0477 mmol/L;

Butyric acid: 13.134 min, LOD=0.1599, LOQ=0.0480 mmol/L;

Isovaleric acid: 14.042 min, LOD=0.1108, LOQ=0.0333 mmol/L;

Valeric acid: 15.612 min, LOD=0.1080, LOQ=0.0324 mmol/L;

Crotonic acid: 16.535 min.
