# Supplementary material for: An in vitro evaluation of the effects of different statins on the structure and function of human gut bacterial community
Source: PLoS One. 2020 Mar 26;15(3):e0230200. doi: 10.1371/journal.pone.0230200 (PMC7098552; doi:10.1371/journal.pone.0230200)

S2 Fig Chromatograms and standard curves of statins.

Standard sample of DMSO: 2.797 min

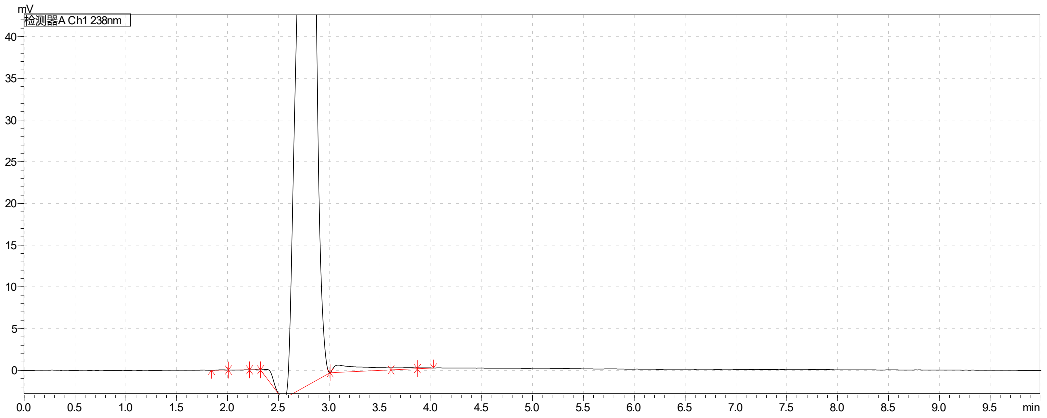

Standard sample of ROS (4.139 min; LOD=0.0143; LOQ=0.048 μg/mL)

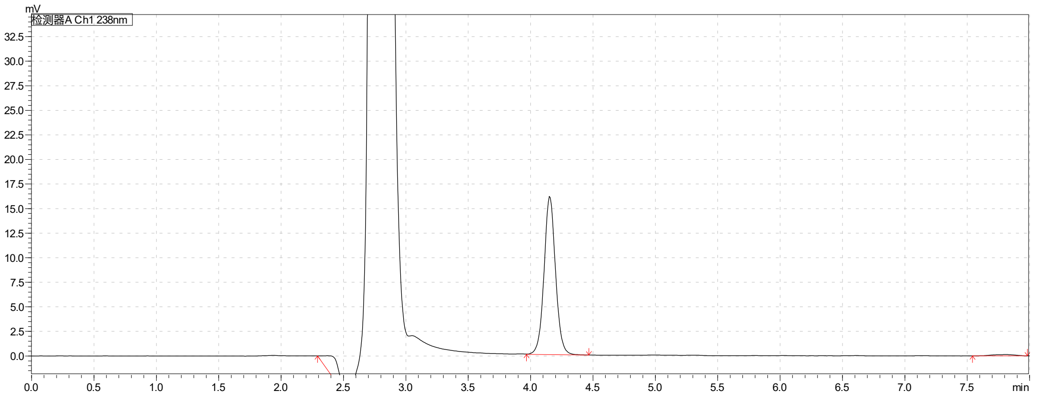

Standard sample of ATO (6.117 min; LOD=0.038; LOQ=0.124 μg/mL)

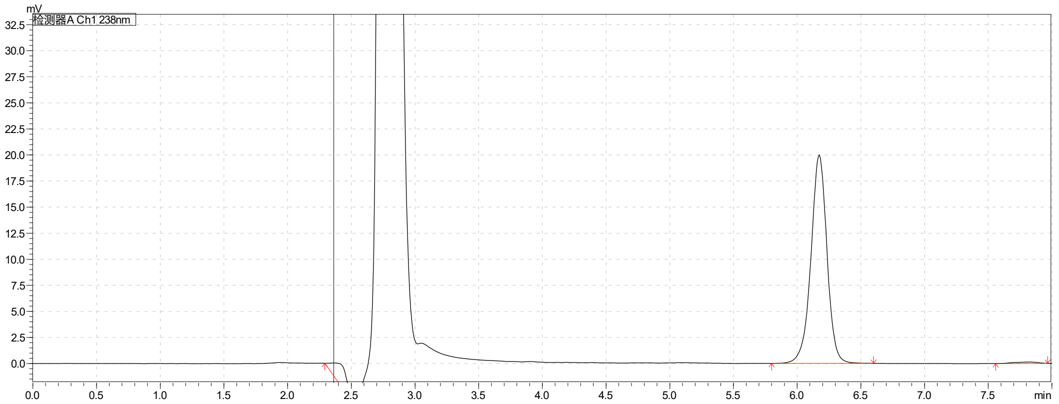

Standard sample of FLU (6.571 min; LOD=0.018; LOQ=0.06 μg/mL)

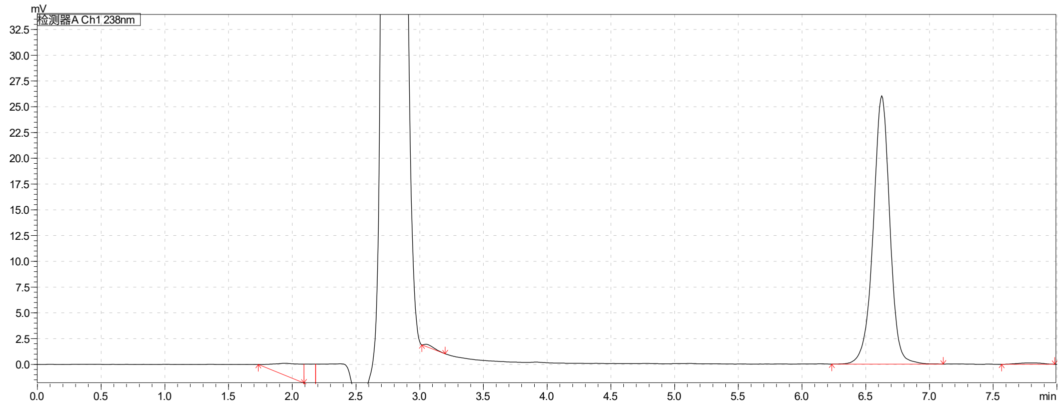

Standard sample of SIM (19.118 min; LOD=0.168; LOQ=0.560 μg/mL)

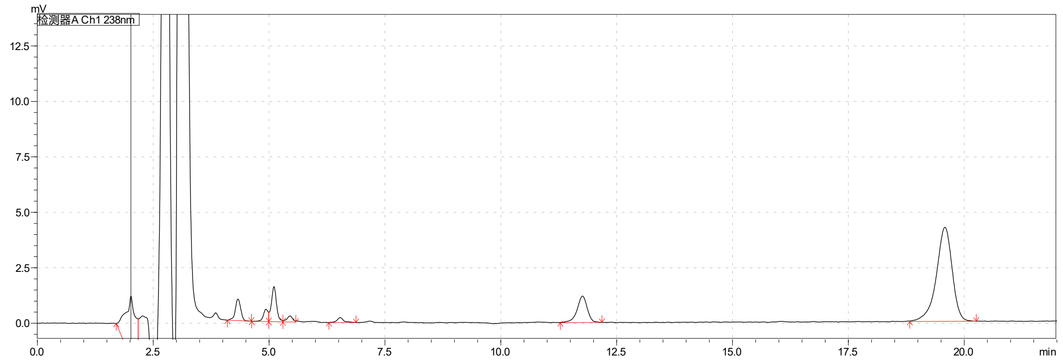

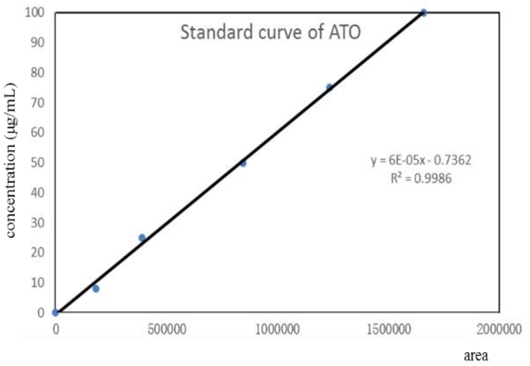

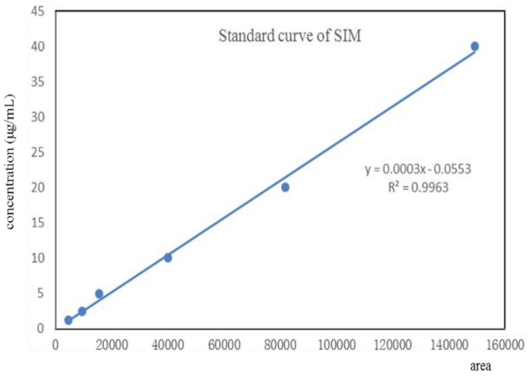

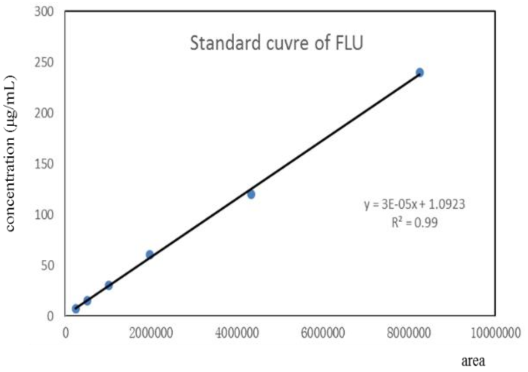

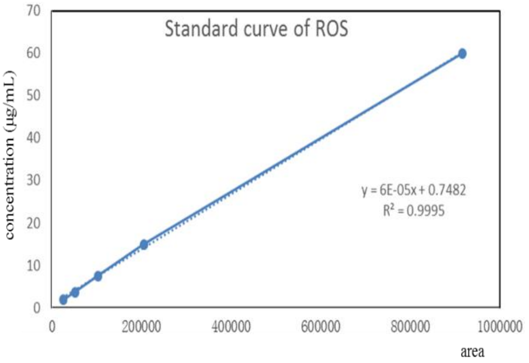

Supplement: S2 Fig — (PDF) [file pone.0230200.s003.pdf]
