# Supplementary material for: An in vitro evaluation of the effects of different statins on the structure and function of human gut bacterial community
Source: PLoS One. 2020 Mar 26;15(3):e0230200. doi: 10.1371/journal.pone.0230200 (PMC7098552; doi:10.1371/journal.pone.0230200)

S3 Fig Basic analysis of sequenced data.

A, Rarefaction curves of sequenced samples. B, The Shannon-Wiener curves of the sequenced samples. C, Rank-abundance distribution curve of the sequenced samples. D, Specaccum analysis of the sequenced samples.

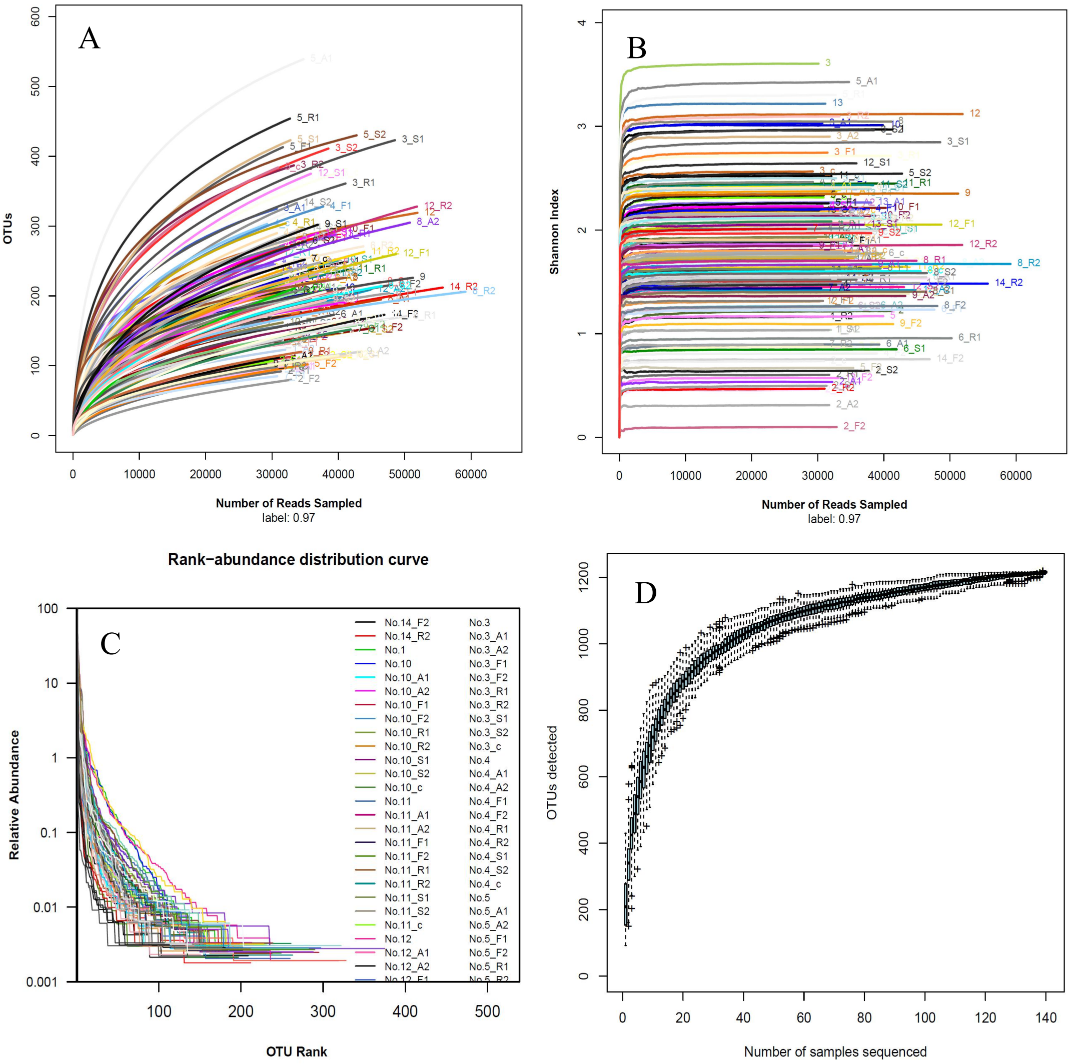

Supplement: S3 Fig — (PDF) [file pone.0230200.s004.pdf]
