# Supplementary figures and images for: An in vitro evaluation of the effects of different statins on the structure and function of human gut bacterial community
Source: PLoS One. 2020 Mar 26;15(3):e0230200. doi: 10.1371/journal.pone.0230200 (PMC7098552; doi:10.1371/journal.pone.0230200)

**S4 Fig LEFSe analysis of different abundant bacterial taxa between groups**  
**ATO2 and control.**

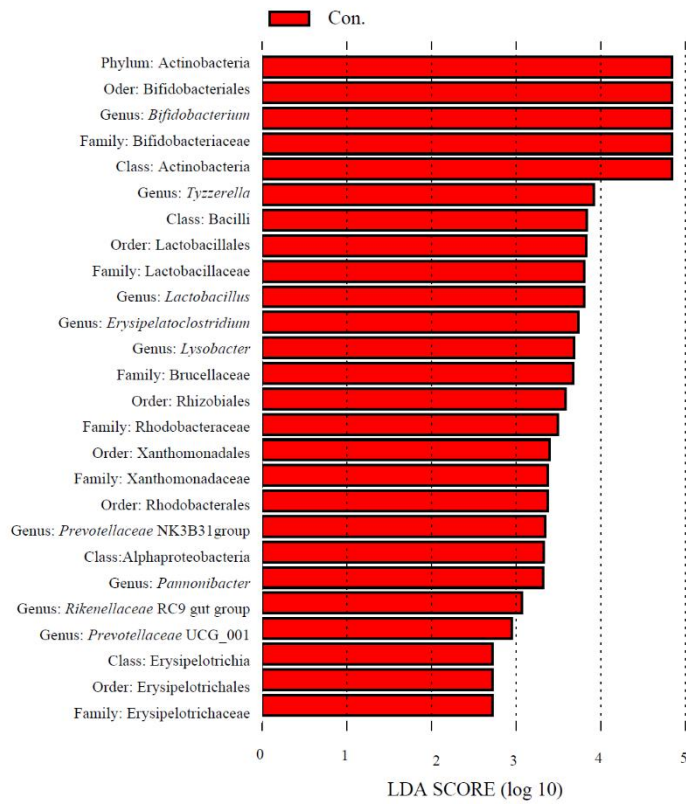

Supplement: S4 Fig — (PDF) [file pone.0230200.s005.pdf]
