# Supplementary material for: An in vitro evaluation of the effects of different statins on the structure and function of human gut bacterial community
Source: PLoS One. 2020 Mar 26;15(3):e0230200. doi: 10.1371/journal.pone.0230200 (PMC7098552; doi:10.1371/journal.pone.0230200)

S5 Fig Bacterial community abundance at the genus level.

No.1–No.14 represents bacteria collected from volunteers. Con, A, S, R, and F represent the control, ATO, SIM, ROS, and FLU groups, respectively; 1 means 20 μM; 2 means colon concentration.

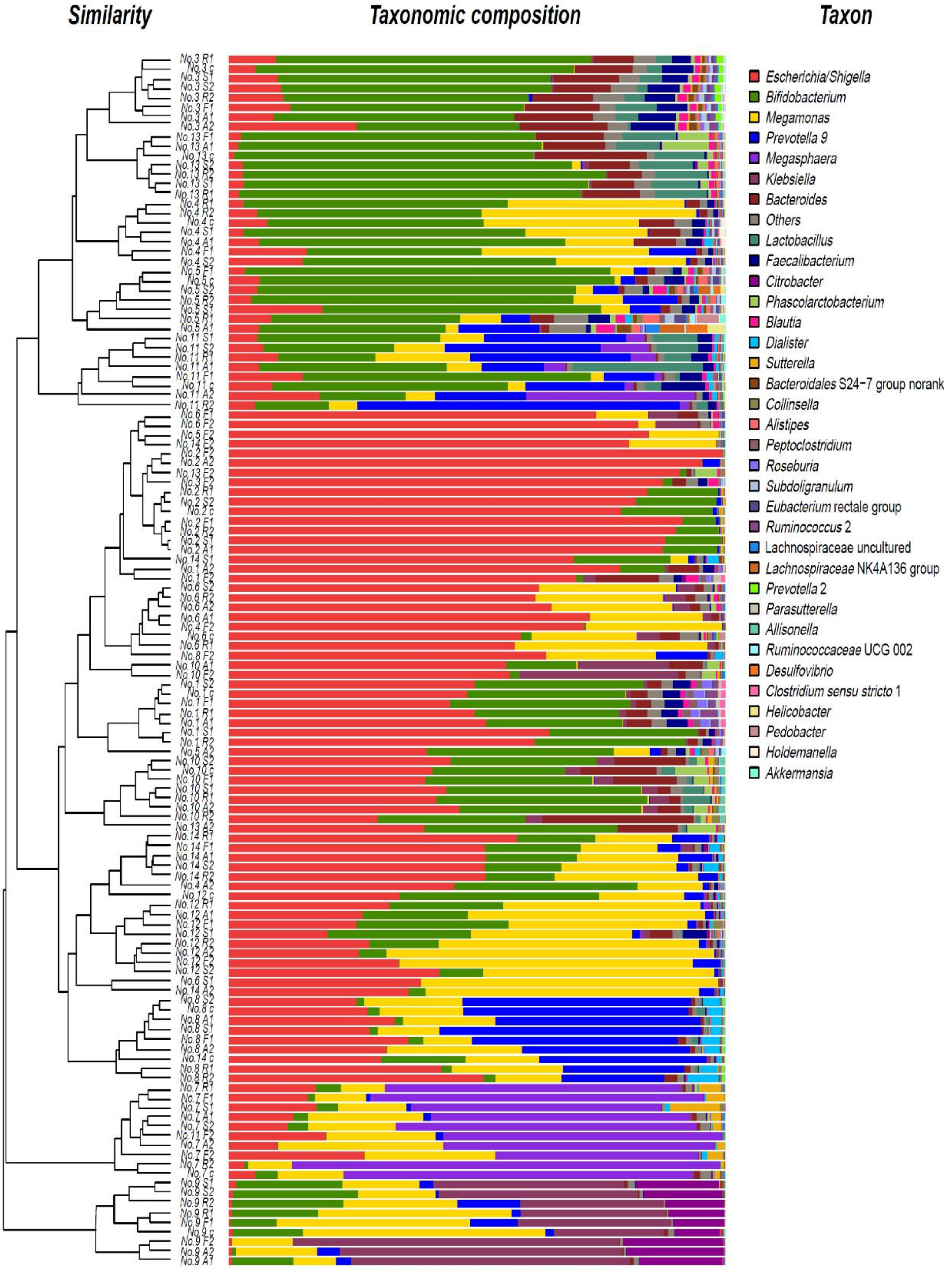

Supplement: S5 Fig — (PDF) [file pone.0230200.s006.pdf]
